# Fully-automated CT derived body composition analysis reveals sarcopenia in functioning adrenocortical carcinomas

**DOI:** 10.1038/s41598-024-62431-2

**Published:** 2024-05-28

**Authors:** Prasanna Santhanam, Roshan Dinparastisaleh, Karteek Popuri, Mirza Faisal Beg, Stanley M. Chen Cardenas, Amir Hamrahian

**Affiliations:** 1 Division of Endocrinology, Diabetes, and Metabolism, Department of Medicine, Asthma and Allergy Center, Johns Hopkins University School of Medicine, 5501 Hopkins Bayview Circle, Suite 3 B 73, Baltimore, MD 21224 USA; 2 University of Florida College of Medicine, Gainesville, FL 32610 USA; 3 Department of Computer Science, Memorial University of Newfoundland, St. John’s, NL Canada; 4 School of Engineering Science, Simon Fraser University, Burnaby, BC V5A 1S6 Canada

**Keywords:** Cancer, Endocrine system and metabolic diseases, Biotechnology, Cancer, Diseases, Endocrinology, Health care, Medical research, Signs and symptoms, Computational biology and bioinformatics, Machine learning

## Abstract

Determination of body composition (the relative distribution of fat, muscle, and bone) has been used effectively to assess the risk of progression and overall clinical outcomes in different malignancies. Sarcopenia (loss of muscle mass) is especially associated with poor clinical outcomes in cancer. However, estimation of muscle mass through CT scan has been a cumbersome, manually intensive process requiring accurate contouring through dedicated personnel hours. Recently, fully automated technologies that can determine body composition in minutes have been developed and shown to be highly accurate in determining muscle, bone, and fat mass. We employed a fully automated technology, and analyzed images from a publicly available cancer imaging archive dataset (TCIA) and a tertiary academic center. The results show that adrenocortical carcinomas (ACC) have relatively sarcopenia compared to benign adrenal lesions. In addition, functional ACCs have accelerated sarcopenia compared to non-functional ACCs. Further longitudinal research might shed further light on the relationship between body component distribution and ACC prognosis, which will help us incorporate more nutritional strategies in cancer therapy.

## Introduction

Adrenocortical Carcinoma (ACC) is a highly aggressive tumor with an annual incidence of 0.5–2 per million people^1^. In about 60% of patients, the diagnosis of ACC is suspected based on clinical and biochemical features of cortisol and androgen excess (functioning ACC). In those that are not associated with hormonal excess (non-functioning ACC), symptoms associated with tumor mass are usually the presentation. These clinical features along with an adrenal mass with radiological features (large, irregular, heterogenous with hemorrhage, necrosis, density > 20HU on unenhanced images on CT, local, and/or extra-adrenal invasion), are typical. However, the diagnosis cannot be established by fine needle aspiration (FNA), as differentiation of malignant versus benign primary adrenal mass is not possible via cytology. In fact, FNA in this setting is contraindicated given the risk for tumor seeding. Accordingly, a surgical specimen is required where the Weiss Scoring system can be applied to establish the diagnosis of ACC^2^.

Guidelines recommend en-bloc open surgery for resectable tumors by an expert surgeon and a multi-disciplinary approach (involving chemotherapy, immunotherapy, stereotactic radiation, and surgery) for advanced and recurrent disease^3,4^. Due to the infrequent incidence of ACC, data are limited regarding factors that could predict disease-free and overall survival^3,4^. Although cachexia can be observed in patients with advanced ACC, no data is available on how this could impact the course of this disease in particular.

Cachexia is characterized by progressive loss of body weight, including skeletal muscle mass or sarcopenia, which is due to systemic inflammation, and cannot be completely reversed by conventional nutritional support^5^. Criteria for diagnosis include weight loss greater than 5% in the past 6 months; body mass index (BMI) < 20 kg/m^2^ and any weight loss > 2%; or an appendicular skeletal muscle index consistent with sarcopenia and any degree of weight loss > 2%^5^. Cachexia afflicts a large group of cancer patients, and malnutrition is a known factor that impacts patients’ response to treatment by increasing treatment-related toxicities, worsening their quality of life, and their overall survival^6,7^.

The presence of cachexia in cancer patients has been shown to be an independent predictor of shorter survival, indicating its role as a prognostic factor for mortality^8–10^.

Given that the overall survival is known to be poor in ACC, with 5-year survival rates estimated at 30–40%, and literature regarding cancer-related cachexia demonstrating mortality of 20–80%, understanding of the role of cachexia and sarcopenia in ACC related mortality becomes very relevant^11^. The main goal of our study is to describe the body composition characteristics in patients with adrenocortical carcinoma applying a highly efficient AI based analytical technique using CT images. Currently, literature lacks application of tools that are automated (using minimal human effort), and integrate advanced AI methodologies with radiological imaging to assess these parameters accurately. Our study addresses this gap by utilizing a highly efficient AI-based analytical technique to analyze CT images for detailed body composition analysis.

We hypothesize that the fully automated CT derived body composition analysis can detect efficiently differences in body composition (muscle, subcutaneous and visceral fat) i.e. cachexia, sarcopenia in the setting of adrenal cortical carcinomas and this information could provide vualuable clinical information about the disease.

## Methods

We analyzed data from the Cancer Imaging Archive (TCIA) and compared the result to a randomly selected group of control participantsfrom the Johns Hopkins Medical Institutions^12^. The cancer imaging archive is a deidentified imaging dataset provided by the NCI. NCI has previously collaborated with Washington University in Saint Louis and created (TCIA), an open source/access imaging database that supports research using advanced imaging of cancer^12^. The TCIA supports both quantitative and qualitative imaging research platforms^13–15^. In TCIA, each image set belonging to a specific participant is linked to a unique identification number associated with a specific clinical presentation (age, sex, disease stage, prognosis, etc.). We analyzed ACC data from the TCIA. The dataset and the relevant publication are cited, as per the datasharing regulations^16–18^.

The inclusion criteria for the original study are as follows^16^:

- Pathologically proven Adrenocortical carcinoma.
- Patients who underwent surgical resection of the tumor.
- Determination of the Ki-67 index as part of the histopathological evaluation of the resected tissue.
- Availability of imaging data, specifically pre-resection contrast-enhanced CT scans of the abdomen.

The exclusion criteria:

1. Data from patients whose Ki-67 index was quantified using biopsied tissue samples rather than from resected whole tumors.

The image dataset was linked to the patient-specific data.The baseline variables, including age, sex, size of the tumor, functionality, and mode of presentation, were retrieved and concatenated. Unfortunately, the individual-level data regarding hormonal levels such as androgens (DHEA-S, androstenedione, testosterone), and cortisol hypersecretion (1 mg dexamethasone suppression Test, 24 h urinary free cortisol levels, and midnight salivary cortisol levels) were not available. However, the mode of presentation has documented the functionality (secretory nature) of the tumors.

The studies concerning the TCIA and the controls (based on chart review without any specific selection criteria, that was obtained from the Johns Hopkins Medical Institutions) were approved by the Johns Hopkins IRB (IRB00154372-12/11/17, IRB00409759 10/24/23). The CT scan images were deidentified and analyzed using automated software.

### Automation technology

CT body composition was performed using the data analysis tool (DAFS) developed by Voronoi Health Analytics Ltd^19–21^. DAFS performed non-linear image processing (using automation algorithms) and provided multi-slice segmentation of tissues and automated vertebral level annotation across different axial slices. DAFS creates an automated workflow by first creating a curated DICOM folder that sorts and creates individual files using embedded information. Then it performs annotation and segmentation (after removing private health information identifiers) and publishes the images and reports in an output folder. A population and scan-wise break-up data is provided in a CSV file for user perusal. Supplementary file (S1) shows the automation process.

The DAFS has been validated to a high degree of accuracy -dice coefficients achieved were 0.980 for bone, 0.974 for skeletal muscle, 0.986 for SAT, and 0.960 for VAT, demonstrating the validity of whole-body 3D tissue segmentation algorithm^22^. Skeletal muscle, visceral adipose tissue, subcutaneous adipose tissue, and intramuscular adipose tissue were segmented and measured from L1–L5 vertebral level cross-sectional area^22^. Measurements were derived from the third lumbar vertebrae (L3) level using the L3 mid-slice automatically identified by the DAFS program and the total of L1–L5 across axial levels. Specific measurements included the tissue compartments of (1) skeletal muscle (with and without psoas muscle), (2) Total adipose tissue comprising subcutaneous adipose tissue, intermuscular adipose tissue, and visceral adipose tissue. L3 level specific and averages (across axial slices) and L1–L5 were computed. The skeletal muscle or the total fat volume was computed by multiplying the area by the slice thickness (W × L × H). The results of the body composition metrics were compared between ACC and non-malignant adrenal lesions, as well as between functional and Non-functional ACCs.

### Ethical compliance and data utilization

The analysis was performed in strict accordance with the ethical standards and policies outlined by Nature Scientific Reports (available at https://www.nature.com/srep/journal-policies/editorial-policies#experimental-subjects). A deidentified, publicly available dataset, acquired several years prior to this research, was utilized. Given the retrospective and anonymized nature of the data, informed consent was deemed not applicable. The Johns Hopkins Institutional Review Board (IRB) approved the research protocol and granted a formal waiver for the requirement of informed consent, recognizing that the research could not be practicably carried out otherwise. The software used in our analysis is detailed herein with version numbers and access URLs to ensure reproducibility (https://www.voronoihealthanalytics.com/dafs). All procedures contributing to this work comply with the ethical guidelines of the relevant national and institutional committees on human experimentation and with the Helsinki Declaration of 1975, as revised in 2013.

### Statistics

After the assimilation of multicentric heterogeneous data, the characteristics of the individual participants were analyzed. A t-test was used to compare continuous variables (age, tumor size) between functional and nonfunctional (classes) ACCs. The chi-square test was used to compare the distribution of categorical data (Metastasis at presentation, Gender, TNM stage) between the two classes. The body composition metrics were compared using the student t-test. Equal variances were not assumed. P- values were considered significant at a threshold α level of 0.05. The SPSS 21 was used for statistical analysis.

## Results

There were nine non-malignant adrenal control participants that were used to compare the different body composition metrics, with the ACC participants (consistent of 41 patients). Among the control participants, there were cases of primary aldosteronism (3/9), nonfunctional adenoma (4/9), and pheochromocytoma (2/9). All the patients were analyzed before surgical intervention.

The mean age was not different between the controls and the ACC cases (52.3 (± 14.5) vs 53.5(± 13.6), p = 0.8). The participants with non- malignant adrenal lesions (controls) had significantly greater skeletal muscle cross-sectional area (at both L1–L5 and the average of L3). Although there was a trend toward greater fat mass in non-malignant adrenal lesions, it did not reach statistical significance. Non-functional adrenal lesions (after excluding pheochromocytoma and primary aldosteronism which may be associated with loss of fat and muscle mass) had higher fat and muscle mass compared to ACCs (not statistically proven due to a smaller sample of the control population). The comparison between non-malignant adrenal lesions and ACCs is shown in Table 1.

**Table 1:** Body composition measures in non-malignant adrenal tumors and adrenocortical carcinoma (ACC).

Group statistics	Adrenocortical carcinoma			Non-malignant adrenal tumors			P-value
Body composition metric	N	Mean	SD	N	Mean	SD	
Skeletal muscle cross sectional area average L1–L5 (cm^2^)	34	101.89	23.11	9	136.57	57.21	** < 0.01**
Skeletal muscle volume L1–L5 (cm^3^)	34	1355.26	344.33	9	1528.52	668.50	0.29
Skeletal muscle cross sectional area L3 average (cm^2^)	39	109.75	24.93	9	145.01	59.96	** < 0.01**
Skeletal muscle volume L3 average (cm^3^)	39	36.05	19.01	9	36.22	31.94	0.9
Total fat cross sectional average area L1–L5 (cm^2^)	34	392.19	197.22	9	503.73	170.75	0.13
Total fat volume L1–L5 (cm^3^)	34	5196.58	2669.94	9	6324.11	3539.51	0.30
Total fat cross sectional area L3 average (cm^2^)	39	402.79	204.45	9	533.93	182.90	0.08
Total fat volume L3 average (cm^3^)	39	119.84	77.81	9	142.80	131.20	0.49

Significant values are given in bold.

There were 26 non-functional and 15 functional ACCs in the cohort. The distribution is outlined in Table 2. There were more functional ACCs that presented at an advanced stage and had metastasis at presentation (33.3% (5/15) vs 0.0% (0/26), p < 0.02). Functional ACCs have significantly lower skeletal muscle content (Skeletal Muscle Average Cross Sectional Area L1–L5 [cm^2^], Skeletal Muscle Volume L1–L5 [cm^3^], Skeletal Muscle Average Cross Sectional Area L3 [cm^2^]) in comparison to non-functional ACCs (p-value < 0.05). Details of the results are outlined in Table 3. There was no difference in total fat cross-sectional area/volume distribution within the same vertebral levels. Tumor size was relatively smaller in functional ACCs, although it did not reach the level of significance. There was no correlation between age or tumor size and the metrics of skeletal muscle content either in functional or non-functional ACCs.

**Table 2:** Baseline characteristics of the adrenocortical carcinoma cohort according to function.

Parameters	Non-functional ACC (N = 26)	Functional ACC (N = 15)	P-value
Age (years)	54.0(± 13.8)	52.1 (± 12.9)	0.6
Tumor size (cm)	13.5(± 7.8)	9.6 (± 4.6)	0.08
Sex			
Male	11(42.3)	4(26.7)	0.317
Female	15 (57.7)	11(73.3)	
Race			
Asian	2 (7.7)	0 (0)	–
Black	3 (11.5)	1 (6.7)	
Latino	3 (11.5)	3 (20.0)	
White	18 (69.2)	11 (73.3)	
TNM stage			
1	3 (11.5)	0 (0)	0.01
2	10 (38.5)	3 (20.0)	
3	13 (50.0)	7 (46.7)	
4	0 (0.0)	5 (33.3)	
Metastasis at presentation	0 (0)	5 (33.3)	< 0.01

Continuous variables (age and tumor size) expressed in mean (± standard deviation).

Categorical variables (sex, race, TNM stage and metastasis at presentation) expressed in number (percentage distribution within the category).

**Table 3:** Body composition measures in non-functional and functional adrenocortical carcinoma (ACC).

Group statistics	Non-functional ACC			Functional ACC			P-value
Body composition metric	N	Mean	SD	N	Mean	SD	
Skeletal muscle cross sectional area average L1–L5 (cm^2^)	22	107.24	26.08	12	92.09	11.94	**0.01**
Skeletal muscle volume L1–L5 (cm^3^)	22	1438.90	385.93	12	1201.90	178.06	**0.01**
All skeletal muscle volume L1–L5 (including Psoas) (cm^3^)	23	1668.57	487.83	12	1494.69	436.20	**0.3**
Skeletal muscle cross sectional area L3 average (cm^2^)	26	119.46	29.76	15	99.51	12.98	** < 0.01**
Skeletal muscle volume L3 average (cm^3^)	26	43.67	20.79	15	26.06	14.65	** < 0.01**
All skeletal muscle volume at L3 (including Psoas)	23	48.83	23.16	12	37.19	26.12	**0.18**
Total fat cross sectional average area L1–L5 (cm^2^)	22	383.75	219.62	12	407.66	155.58	0.36
Total fat volume L1–L5 (cm^3^)	22	5076.51	2862.51	12	5416.72	2379.28	0.36
Total fat cross sectional area L3 average (cm^2^)	26	402.13	235.19	15	393.97	165.26	0.45
Total fat volume L3 average (cm^3^)	26	131.54	80.96	15	95.17	68.00	0.07

Significant values are given in bold.

**Figure 1:**
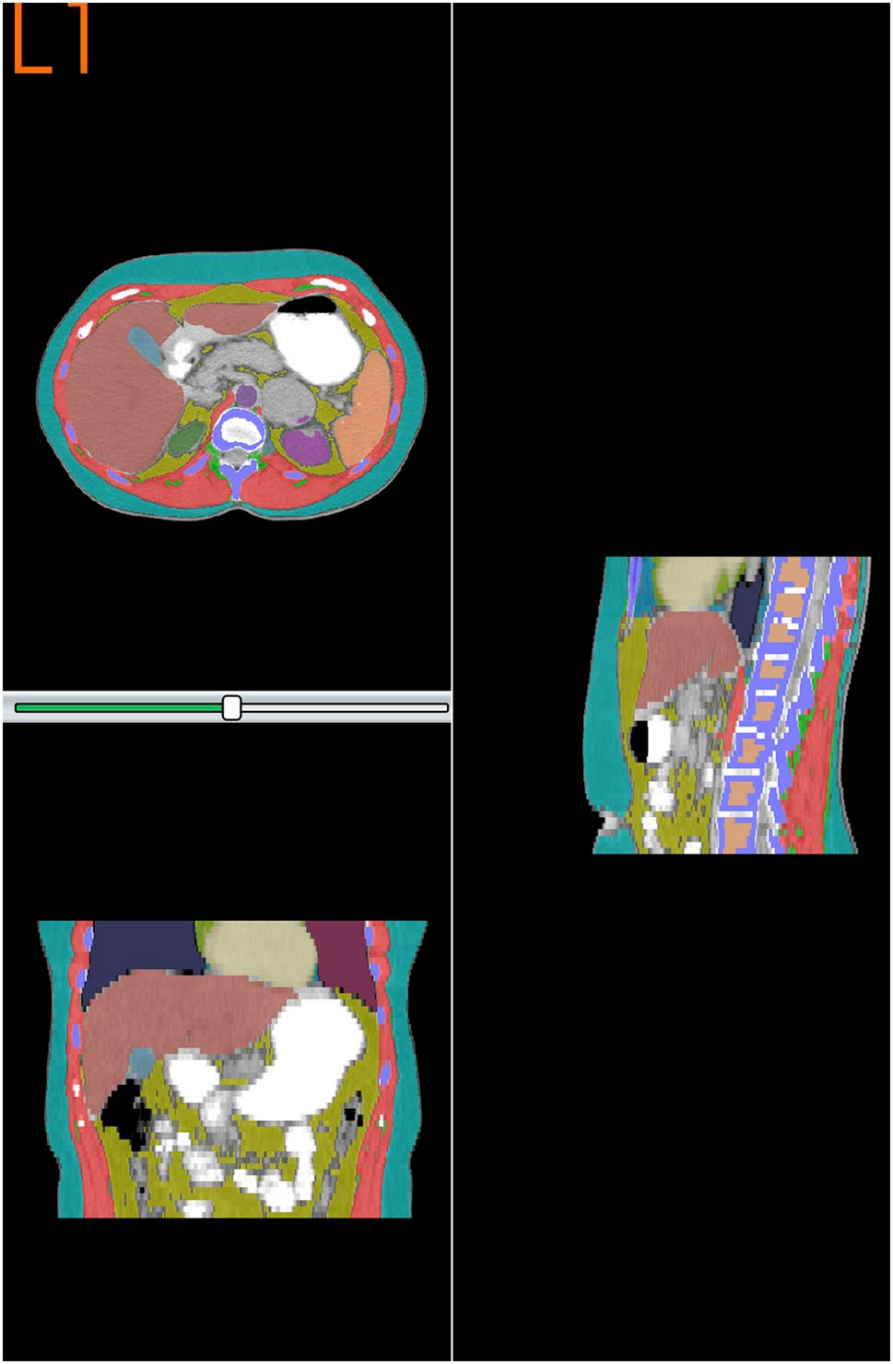
Axial CT scan image at L1 with automated body composition in a 35-year-old woman with ACC.

**Figure 2:**
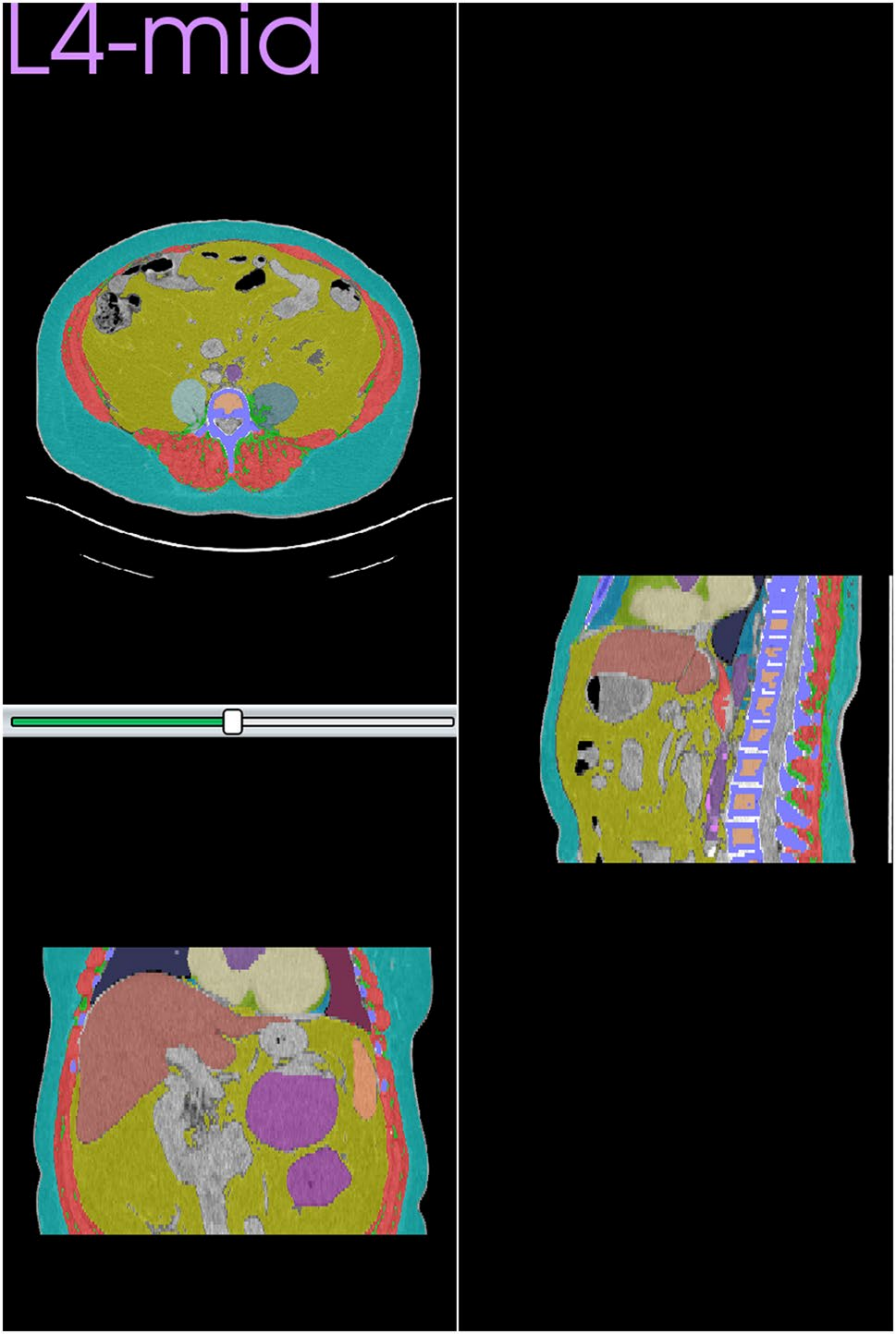
Axial CT scan image at L4 mid with automated body composition in a 53-year-old woman with ACC.

**Figure 3:**
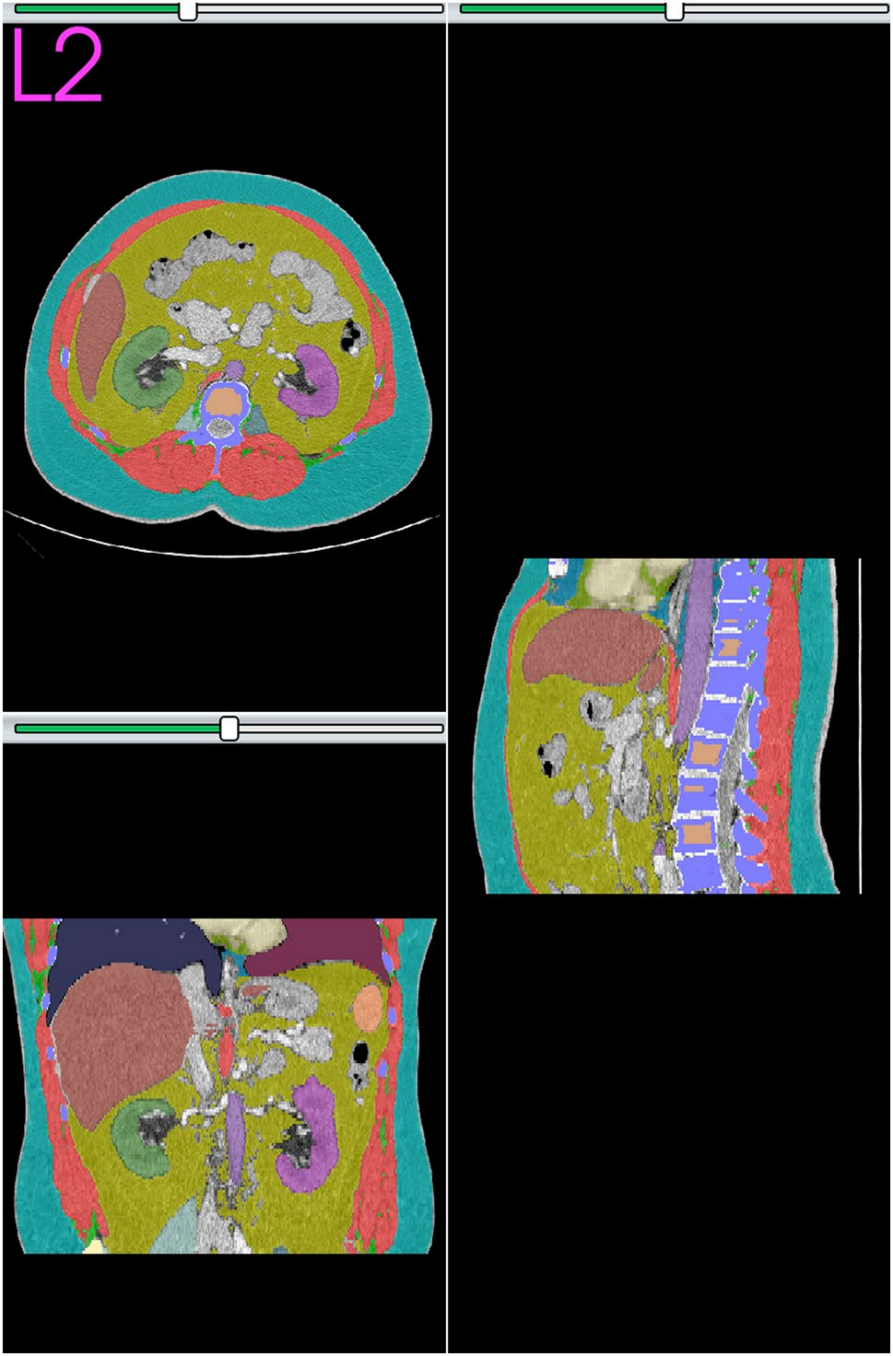
Axial CT scan image at L2 mid with automated body composition in a 53-year-old man with ACC.

Figures 1, 2 and 3 show the automated segmentation images obtained from three cases of ACC (figure legends are outlined below). The labeling is as follows: VAT—yellow; intermuscular adipose tissue in green; Skeletal Muscle—Red; and SAT (subcutaneous adipose tissue) in blue. ACC is shown as the large gray lesion.

## Discussion

Sarcopenia, or muscle loss, is linked to worse outcomes in various cancers. In a study involving colorectal cancer patients, sarcopenia was shown to lower disease-free survival, overall survival, and cancer-specific survival^25^. In patients with adenocarcinoma at the esophagogastric junction and upper gastric cancer, development of sarcopenia after surgery predicted a poor prognosis, emphasizing the need to monitor body composition (especially muscle mass changes) both before and after treatment^26^. Additionally, in patients with gastric cancer who are treated with PD-1 inhibitors, sarcopenia is associated with shorter progression-free survival, though its impact on overall survival is not significant^27^. All these studies have underscored the importance of quantification of muscle mass loss in advanced and aggressive malignancies.

In this research study, we have used a novel automated body composition measuring algorithm to assess body composition in patients with adrenocortical carcinomas. Application of artificial intelligence (AI) to medicine by use of various machine and deep-learning algorithms has been constantly evolving. It is soon likely to be the standard of care going forward^28^. Serial CT Abdomens (as a part of regular CT with or without contrast use and with a radiotracer based (FDG- PET) are performed for the staging and prognosis of advanced malignancies, but vital biomedical information vis a vis body composition are under-reported/under-utilized for prognostication. CT body composition was performed using the data analysis tool (DAFS) developed by the co-authors. DAFS has been reported and shown to be highly accurate in different malignancies such as esophageal cancer and lymphomas^20,21^. The use of a fully automated algorithm for assessing an endocrine-related malignancy is a novel use and application of this technology.

A previous study showed that ACC that secreted mixed cortisol and androgens had a worse prognosis than either cortisol or androgen alone, if adequate resection was not achieved in the initial surgery^29^. In this large multicenter study, increasing age, higher Ki67%, and mixed secreting phenotype were associated with poor prognosis^29^.

Our study shows relative sarcopenia in functional ACC compared to both nonfunctional ACCs and non-malignant adrenal lesions. A recently published study, evaluating 35 patients with ACC showed that sarcopenia, assessed by calculating the skeletal muscle index was associated with a shorter overall survival^30^. This study was performed using core-slicer (a semi-automation technology) that requires extensive manual effort and maybe associated with interoperator variability. The skeletal muscle cross sectional area was measured at a single level (L3), and the index was calculated at that level. It was concluded that sarcopenia correlates with shorter survival and that the influence of hormonal status needed to be further examined^30^. Another study reported an increase in sarcopenia and intra-abdominal fat correlated with decreased survival in patients with ACC^7^. However, the study only specifically measured the psoas muscle, and the functionality of the ACC was not part of the analysis. Our results are consistent with other methods of body composition measurements that are more laborious and time intensive.

Here, we show that the functionality of the ACC further worsens skeletal muscle density. We have used a fully automated technology that measures the cross-sectional area at various levels (L1–L5) and shown that there is indeed profound loss in muscle mass at diverse levels compared to non-functional ACCs and other adrenal neoplasms. Although we do not have the details of hormonal evaluation for the cases, the patients presented with hormonal functionality. It is unclear if active interventions to improve muscle mass by enhancing exercise and protein intake, concomitantly with medical and surgical management, improve progression-free and overall survival. Some ACC patients have limitations in their ability to exercise coupled with a diminished appetite secondary to concomitant medications such as mitotane and chemotherapy or the tumor itself, limiting the potential success of these interventions.

The strength of our study includes the employment of a publicly available cancer imaging with a diverse/heterogeneous study population under different clinical settings, the use of automated technology, and the comparative analysis with nonmalignant etiologies. We relied on the designation of functional ACC based on the dataset. The main limitation as indicated previously, given the lack of available information, is that we could not establish the extent of the influence of glucocorticoid or androgen excess in the radiological findings observed. However, we consider that our data, generated using an efficient automated technique provide interesting insight on the anatomical effects of functional ACC and prognosis.

Variations in scan protocols across studies might result in heterogeneity in data and image quality between different data sources. The study utilizes data from a public archive and a specific academic center, which may not represent the broader ACC patient population and might represent a selection bias. However, studies in ACC are generally multicentric and heterogenous since the condition is not very common. Finally, while the findings suggest potential applications in clinical practice, the actual integration of these automated body composition measures into routine clinical workflows and their impact on patient management require further validation through prospective multicentric clinical trials.

In summary, fully automated CT derived body composition metrics offer insights into the behavior of ACC. Automated CT-derived muscle and fat mass estimation might help in prognostication of ACC after duly long-term prospective studies are conducted in this regard.

## Supplementary Information

Supplementary Information.

## Data Availability

The data that support the findings of this study are available from TCIA (The Cancer Imaging Archive) and the Johns Hopkins Hospital but restrictions apply to the availability of these data, which were used under license for the current study, and so are not publicly available. Data are however available from the authors upon reasonable request and with permission of the above mentioned entities.
