# Supplementary material for: Fully-automated CT derived body composition analysis reveals sarcopenia in functioning adrenocortical carcinomas
Source: Sci Rep. 2024 May 28;14:12193. doi: 10.1038/s41598-024-62431-2 (PMC11133428; doi:10.1038/s41598-024-62431-2)
Supplement: Supplementary file 1 — Supplementary Information. [file 41598_2024_62431_MOESM1_ESM.docx]

**Summary of the technology in 4 Steps**

**DICOM Curation:** DAFS initiates its workflow by curating DICOM files. It automatically detects and organizes DICOM files from an input folder, sorts them based on embedded metadata, and segregates each unique scan into its own folder within a designated output folder. This process is essential for ensuring the integrity and organization of the data before any further analysis or processing is performed.

**Anonymization and Segmentation**: DAFS incorporates a robust anonymization feature that automatically removes or preserves specified DICOM attributes to protect patient privacy. Following anonymization, the system offers advanced segmentation capabilities. It can differentiate and segment various anatomical and pathological features within the scans using an AI-driven engine, which is adjustable in terms of the Hounsfield Unit (HU) range for precise imaging analysis.

**Annotation and Quickchecks**: The system provides tools for annotating medical images with a focus on specific slices or regions, facilitating detailed study of particular anatomical landmarks. Additionally, 'Quickchecks' are generated to provide visual feedback and quality assurance, showcasing the accuracy of segmentations and annotations on the scans, which is crucial for reliable medical analysis.

**Publication and Reports:** Finally, DAFS compiles the processed and analyzed data into comprehensive reports and CSV files, which can be customized to include specific DICOM attributes or results from the segmented and annotated data. This functionality is critical for researchers and clinicians to derive meaningful insights and conclusions from the imaging data.

This suite is particularly designed to enhance the efficiency and accuracy of medical imaging studies, providing a streamlined, automated workflow that leverages AI technologies for improved diagnostic and research outputs. For detailed information about the underlying algorithms or to seek further assistance, interested parties are encouraged to contact Voronoi Health Analytics through their website at https://www.voronoihealthanalytics.com/.
